# The Role of Caspase-3, Apoptosis-Inducing Factor, and B-cell Lymphoma-2 Expressions in Term Premature Rupture of Membrane

**DOI:** 10.1055/s-0038-1675611

**Published:** 2018-12

**Authors:** Ketut Surya Negara, Ketut Suwiyoga, Tjokorda Gede Astawa Pemayun, Anak Agung Raka Sudewi, Nyoman Mantik Astawa, I Gusti Nyoman Kamasan Arijana, Ketut Tunas

**Affiliations:** 1 Department of Obstetrics and Gynecology, Medical Faculty of Udayana University /Sanglah Hospital, Bali, Indonesia; 2 Department of Neurology, Medical Faculty of Udayana University/Sanglah Hospital, Bali, Indonesia; 3 Laboratory of Veterinary Medicine, Udayana University, Bali, Indonesia; 4 Biomedic Laboratory of Medical Faculty, Udayana University, Bali, Indonesia; 5 Department of Public Health, Dhyana Pura University, Bali, Indonesia

**Keywords:** PROM, caspase-3, apoptosis inducing factor, B-cell lymphoma-2 expression, apoptosis

## Abstract

**Objective** To determine the role of caspase-3, apoptosis-inducing factor (AIF), and B-cell lymphoma-2 (Bcl-2) expressions in term premature rupture of membrane (PROM).

**Methods** An analytic observational study with case-control design was conducted, involving 52 subjects (37–42 weeks of gestation) who were divided into 2 groups: 26 cases of term delivery with PROM, and 26 controls of term delivery without PROM. The expressions of caspase-3, AIF, and Bcl-2 in the amniotic membrane were determined by immunohistochemistry. Data were analyzed using the chi-squared test. The risk of PROM was expressed by odds ratio (OR).

**Results** There were no significant differences in age, parity and body mass index between the two groups (*p *> 0.05). High caspase-3 and AIF expressions increased the risk of PROM 17.64 times (OR = 17.64; 95% CI = 4.44–70.07; *p* = 0.001) and 9.45 times (OR = 9.45; 95% CI= 2.62–34.07; *p* = 0.001), respectively, while low Bcl-2 expression increased 10.39 times (OR = 10.39; 95% CI = 2.73–39.56; *p* = 0.001)the risk of PROM .

**Conclusion** High caspase-3 and AIF expressions and low Bcl-2 expression were risk factors for term PROM. Caspase-dependent and independent pathways of apoptosis were involved in the mechanism of PROM in term pregnancy.

## Introduction

Premature rupture of the membrane (PROM) is one of the complications of pregnancy, and it is associated with maternal and neonatal morbidities.^1^ Term PROM is defined as the rupture of the fetal membrane prior to the onset of labor in a term pregnancy. The etiology of PROM is multi-factorial, and its pathobiological mechanisms are unclear.^2^ One of the factors involved in the endogenous and exogenous mechanisms associated with the increased risk of PROM is the occurrence of programmed cell death, or apoptosis.

The incidence of PROM worldwide varies between 5 and10%, and almost 80% of the cases occur at term pregnancy.^1^ ^2^ In China, the reported incidence of PROM is higher, around 19.53% of all pregnancies.^3^ Budijaya et al (2017)^4^ reported 212 cases of PROM in 1,450 deliveries (14.62%) at the Sanglah Hospital Denpasar, Bali, Indonesia; 179 cases (84.43%) were term pregnancies (≥37 weeks), while 33 cases (15.57%) were preterm pregnancies.

Premature rupture of the membrane is associated with various complications for both the mother and the newborns. Maternal complications such as chorioamnionitis are found in 9% of the pregnancies with PROM, and the incidence is greater in preterm pregnancy, reported in between 13 and 60% of these cases.^5^ ^6^ Placental abruption occurs in between 4 and 12% of the pregnancies with PROM. Neonatal complications of PROM, such as intrauterine infection, umbilical cord compression, respiratory distress syndrome (RDS), necrotizing enterocolitis, intraventricular hemorrhage, and sepsis are more common in preterm labor. Overall, PROM is associated with 21.4% perinatal morbidity and with a mortality rate of between 18 and 20%.^7^ ^8^ ^9^

The cause of PROM is multifactorial, and its mechanism is unclear. The weakening of the extracellular matrix in the fetal membrane due to collagen degradation predisposes pregnant women to PROM.^5^ This process is thought to be the result of biochemical remodeling and apoptosis, as well as of membrane stretching, which lead directly to tissue damage.^10^ ^11^ ^12^

Apoptosis plays an integral role in the pathogenesis of PROM. Apoptotic cells are found in the amniotic and chorionic layers and are most abundant at the site of the rupture, known as the paracervical weak zone.^13^ ^14^ ^15^ The process involved in the formation of the paracervical weak zone, in addition to the remodeling process, is closely related to the apoptotic mechanism. Apoptotic cells were found to be more abundant in the amniotic membrane of patients with PROM compared with patients without PROM, while the rate of apoptosis was found to be highest in the vicinity of the cervix compared with the fundal area.^16^ ^17^ ^18^

Apoptosis occurs through two mechanisms: caspase-dependent and caspase-independent pathways. The caspase-dependent pathway can be triggered intrinsically by mitochondrial metabolic failure or extrinsically by death receptor activation. The caspase-independent pathway can be triggered by mitochondrial proteins such as apoptosis-inducing factor (AIF) and endonuclease G (endoG), which are produced by the mitochondrial membrane due to the depolarization of the outer mitochondrial membrane.^19^ ^20^ ^21^

The activation of caspase is the initial step of apoptosis, with caspase-3 as the most important executor caspase. Caspase-3 plays an important role in the morphologic changes of the cells and in the biochemical events related to the implementation and completion of apoptotic processes.^20^ ^22^ When a cell is exposed to infection or to a stressor, apoptosis may occur without involving the classic caspase-dependent pathway. In this state, apoptosis occurs through another mechanism, called caspase-independent pathway, which involves Bax, a member of the B-cell lymphoma-2 (Bcl-2) pro-apoptotic family. Studies have suggested that the role of the Bcl-2 protein family in the caspase-independent apoptotic pathway is by affecting mitochondria, which leads to DNA fragmentation.^22^ ^23^ ^24^ The fact that excessive expression of Bax or Bak proteins can induce cell death without involving caspase indicates that factors other than caspase may also initiate apoptosis. Some of these factors are present in mitochondria, namely AIF, which causes chromatin condensation and the release of cytochrome C in the absence of caspase activation.^20^ ^24^ ^25^

## Methods

This is an analytic observational study with a case-control design involving 52 subjects (37–42 weeks of gestation), who were divided into case and control groups. The study was conducted at the Sanglah Central General Hospital, Bali, Indonesia, and at affiliated educational district hospitals. Cases (*n* = 26) were term pregnancies with PROM, and controls (*n* = 26) were term pregnancies without PROM. Premature rupture of the membrane was defined as the spontaneous rupture of the fetal membrane, at least 1 hour before the onset of labor (regular uterine contractions and cervical dilation or effacement). Patients with obesity, maternal infection, systemic illness, obstetrics complications, smoking habit, and history of preterm PROM in the current pregnancy were excluded.

The deliveries of the subjects were spontaneous or medically-induced. After delivery, the placentas were examined, and the site of rupture was visually identified. The study materials were taken from the edge of the ruptured membranes. The expression of caspase-3, AIF, and Bcl-2 was determined using immunohistochemistry examination, performed at the Integrated Biomedical Laboratory of the Medical Faculty of the Udayana University, Bali, Indonesia. The measurement was done in a semiquantitative fashion; positive expression was defined if ≥10% of the cells were stained. The independent *t*-test was used to analyze the differences in caspase-3, AIF, and Bcl-2 expressions between the two groups. The correlation between caspase-3, AIF, and Bcl-2 expression with PROM was assessed using the chi-squared test and is expressed in odds ratio (OR).

## Results

A total of 52 research samples were collected, comprising 26 samples with term labor with PROM, and 26 samples of term labor without PROM. The statistical test using the independent *t*-test showed that there were no significant differences in age, parity, and body mass index (BMI) between the two groups (*p *> 0.05).

In Table 1, we can see that the mean age of the case group was 27.62 ± 6.23 years old, and in the control group it was 29.65 ± 5.90 years old (*p* = 0.231). The mean parity of the case group was 0.69 ± 0.84, and in the control group it was 1.19 ± 1.06 (*p* = 0.065). The mean BMI of the case group was 25.83 ± 5.28 kg/m2, and in the control group it was 23.63 ± 4.58 kg/m2 (*p* = 0.115). These results showed that there was no significant characteristic difference between the two study groups (*p *> 0.05).

**Table 1:** Distribution of age, parity, and BMI in both groups

Risk Factor	Case group (*n* = 26)		Control group (*n* = 26)		*p*-value
	Mean	SD	Mean	SD	
Age (years old)	27.62	6.23	29.65	5.90	0.231
Parity	0.69	0.84	1.19	1.06	0.065
BMI (kg/m^2^)	25.83	5.28	23.63	4.58	0.115

Abbreviations: BMI, body mass index; SD, standard deviation.

**Fig. 1:**
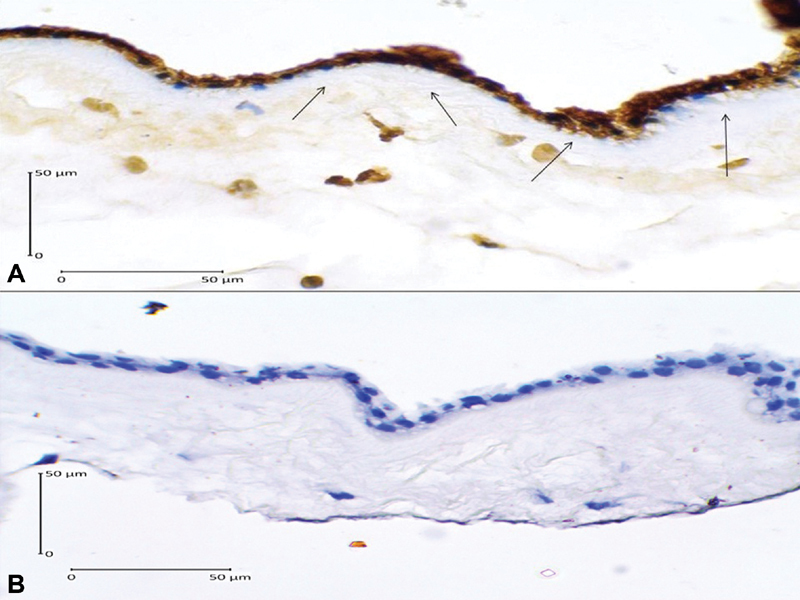
Caspase-3 expression in the amniotic epithelial cells (400x magnification). (**A**) Positive expression shown by dark brown cytoplasm staining with blue nucleus (arrows); (**B**) Negative expression shown by clear cytoplasm with bright blue nucleus.

**Fig. 2:**
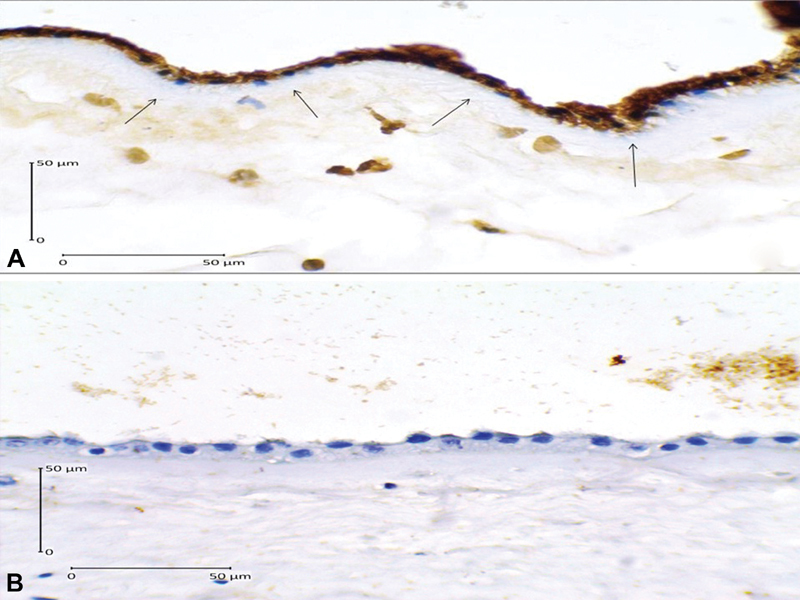
Apoptosis inducing factor expression in the amniotic epithelial cells (400x magnification). (**A**) Positive expression shown by dark brown cytoplasm staining with blue nucleus (arrows); (**B**) Negative expression shown by clear cytoplasm with bright blue nucleus.

**Fig. 3:**
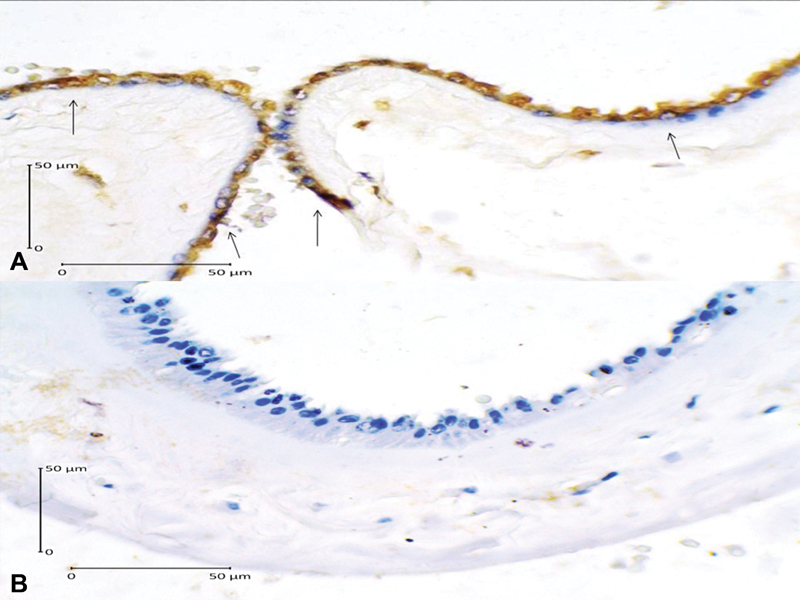
B-cell lymphoma-2 expression in the amniotic epithelial cells (400x magnification). (**A**) Positive expression shown by dark brown cytoplasm staining with blue nucleus (arrows); (**B**) Negative expression shown by clear cytoplasm with bright blue nucleus.

As we can see in Table 2, we have found that high expression of caspase-3 increases 17.64 times the risk of PROM (OR = 17.64; 95% CI = 4.44–70.07; *p* = 0.001), and that high AIF expression increased 9.45 times the risk of PROM (OR = 9.45; 95% CI = 2.62–34.07; *p* = 0.001). The immunohistochemical examination images of caspase-3 and AIF expression are presented in Figs. 1 and 2. We have also found that low expression of Bcl-2 increased 10.39 times the risk of PROM (OR = 10.39; 95% CI = 2.73–39.56; *p* = 0.001). The immunohistochemical examination image of Bcl-2 expression is presented in Fig. 3.

**Table 2:** The expressions of caspase-3, AIF, and Bcl-2 in amniotic epithelial cells at term pregnancy and the risk of PROM

		Group		OR	95% CI	*p*-value
		Case	control			
Caspase -3	High	21	5	17.64	4.44–70.07	0.001
	Low	5	21			
AIF	High	21	8	9.45	2.62–34.07	0.001
	Low	5	18			
Bcl-2	Low	22	9	10.39	2.73–39.56	0.001
	High	4	17			

Abbreviations: AIF: apoptosis inducing factor; Bcl-2, B-cell lymphoma-2; CI, confidence interval; OR, odds ratio; PROM, premature rupture of membrane.

## Discussion

Budijaya et al (2017)^4^ found that the incidence of PROM in term pregnancies (≥37 weeks) was higher than in preterm pregnancies (< 37 weeks): 179 cases (84.43%) and 33 cases (15.57%), respectively. Similarly, Adeniji et al (2013)^1^ reported that most of the cases of PROM (64%) occurred in term pregnancy, while 35.1% of the cases occurred in preterm pregnancy. Another study in India reported that ∼ 82.1% of the PROM cases occurred at term, while 17.6% occurred before term.^12^ Okeke et al (2014)^26^ reported an incidence of 3.3% in preterm pregnancies.

Budijaya et al (2017)^4^ also found that the highest incidence of PROM (41.05%) occurred in primigravida. A similar result was also reported by Noor et al (2007)^27^ and by Gahwagi et al (2015),^28^ who found that the incidence of PROM was also highest in primigravida.^27^ Premature rupture of the membrane was found to be closely related to weight at the time of pregnancy and to a low BMI before pregnancy.^29^

Harirah et al (2012),^14^ in their study about the role of apoptosis in amniotic membranes of patients with term labor, found that the apoptotic index was increased in the chorionic trophoblast at the site of spontaneous ruptures, which was three times higher than in those patients who underwent cesarean section (CS) (artificial rupture). After vaginal delivery, the examination of the choriodecidual layer showed a higher expression of active caspase-3 pro-apoptotic protein and a lower expression of Bcl-2 anti-apoptotic protein, when compared with the CS group. The Bcl-2 protein family is the key regulator of apoptosis, comprising the anti- and pro-apoptotic proteins.^14^

In a study by Xu et al (2005),^13^ it was found that the *CASP-3* gene expression was increased in the amniotic membrane of PROM patients, which indicated an increase in apoptotic cells in the membranes. The increase of apoptotic cells promotes the degradation of the extracellular matrix, leading to decreased membrane elasticity and strength, ultimately causing PROM. Caspase expression and activation play a very important role in apoptosis. They found that caspase-3 expression was higher in patients who had vaginal delivery compared with CS. The immunohistochemical examination revealed that caspase-3 was increasingly expressed in amniotic epithelial cells and in chorionic cytotrophoblast cells, but only slightly expressed in mesenchymal and in reticular cells of the matrix. This finding shows that apoptosis may occur both in the amnion and in the chorion, and plays an important role in the regulation of the fetal membrane.^13^ The role of caspase-3 on preterm PROM was also studied by Saglam et al (2013).^15^ Using a case-control design, they compared the immunopositivity of active caspase-3 in preterm PROM and in preterm delivery with intact membranes (case group) with term pregnancy with normal delivery (control group). It was concluded that the active caspase-3 immunopositivity of the preterm PROM group was significantly higher than that of the control group (*p* < 0.05).^15^ A similar study by Negara et al (2017)^30^ found that positive caspase-3 expression increased 7.3 times the risk of PROM (OR = 7.31; CI 95% = 2.64 to 20.22; *p* = 0.001).

Caspase-3, which is a key factor in the apoptosis execution, is an active form of procaspase-3 and is considered as the most important executor caspase. It is activated by an initiator caspase (caspase-8, caspase-9, caspase-10), which in turn activates caspase-activated DNase (CAD) endonuclease. In proliferating cells, CAD forms a complex with its inhibitor, known as inhibitor of caspase-activated DNase (ICAD). Contrarily, in cells undergoing apoptosis, the activated caspase-3 cleaves ICAD, thus releasing CAD. Caspase-activated-DNase disturbs the chromosomal DNAs inside the nuclei of the cells, causing chromatin condensation. It also triggers the cytoskeletal reorganization and further disintegrates cells to form apoptotic bodies. This process results in phagocytosis.^20^ In our study, caspase-3 expression was significantly higher in the case group compared with the control group (OR = 17.64; 95% CI = 4.44–70.07; *p* = 0.001). This finding suggested that apoptosis via the caspase-dependent pathway was involved in the regulation of the amniotic membrane, and the increased expression of caspase-3 increased the risk of PROM in term pregnancies.

Apoptosis may also occur in a caspase-independent fashion. In this setting, apoptosis is mediated by mitochondrial proapotosis proteins, that is, AIF and endoG.^20^ When a cell receives apoptotic stimulation, AIF and endoG transcend from the mitochondria to the nucleus and stimulate DNA nuclear fragmentation.^20^ ^31^ ^32^ Apoptosis inducing factor is the first protein to mediate caspase-independent cell death. Formerly, AIF was considered as a soluble protein in the intermembranous space of mitochondria, that could be transferred into the nucleus to participate in large-scale DNA fragmentation and chromatin condensation. However, further studies found that AIF is the amino-terminal (N-terminal) and is anchored to the inner mitochondrial membrane. Therefore, AIF must be released from the mitochondrial membrane before it reaches the cytoplasm. It is believed that upon entry into the nucleus, AIF recruits or activates endonucleases to facilitate DNA fragmentation and chromatin condensation.^20^

Apoptosis inducing factor has the capacity to activate independent peripheral caspases and to induce chromatin condensation and DNA fragmentation when specific extracellular signals trigger mitochondrial permeability transition pore (MPTP) opening. This event allows the release of AIF and other apoptogenic effectors, such as apoptosis protease activating factor 1 (APAF-1) and cytochrome C, both of which can activate caspase cascade. Cytosolic AIF triggers the release of more AIF from the mitochondria, forming a self-reinforcing circle that accelerates apoptosis. The proteins of the Bcl-2 anti-apoptosis family serve as protective agents of the mitochondrial membrane to prevent the release of cytochrome C and AIF. In the mitochondrial membrane, the Bcl-2 protein is also involved in regulating the redistribution of AIF in the mitochondrial nucleus.^33^ ^34^ ^35^

In the present study, we have found that high AIF expression increased 9.45 times the risk of PROM (OR = 9.45; 95% CI = 2.62–34.07; *p* = 0.001). This finding suggested that caspase-independent apoptosis was also involved in the mechanism of PROM. Recent studies have shown that some types of cell death may take place in the absence of caspase activation. In some models of cell death, certain caspase inhibitors cannot prevent apoptosis stimulated by pro-apoptotic signals, and caspase activation only is not sufficient to initiate apoptosis. In addition, Bax or Bak expression induces cell death without involving caspase, which indicates that factors other than caspase may also mediate apoptosis. Some of these factors are of mitochondrial origin (such as AIF) and cause mitochondrial swelling, chromatin condensation, and cytochrome C release in the absence of caspase activation.^25^

Cytochrome C release from mitochondria can be triggered by various stress signals originating from the inside of the cell or following caspase activation that is stimulated by surface receptor ligands. The integrity of the outer membrane and the release of cytochrome C from the mitochondria are governed by proteins of the Bcl-2 family, comprising anti-apoptotic factors such as Bcl-2 and B-cell lymphoma-extra large (Bcl-XL), and also pro-apoptotic proteins such as Bax and Bak. These proteins may undergo heterodimerization with each other and interact with the mitochondria, where they play a key role in determining whether the cell will live or die. Thus, Bcl-2 proteins prevent apoptosis by preventing the release of proteins from mitochondrial membranes (i.e. cytochrome C and AIF), while Bax stimulates the release of cytochrome C from mitochondria, resulting in apoptosis.^25^

Obligate intracellular bacterial infection is the most common condition in which the caspase-independent pathway takes action. This is made possible by the direct destructive ability of the bacteria on the mitochondrial membrane, resulting in DNA fragmentation and nuclear chromatin division to produce oligonucleosomal DNA fragments. Biochemical and genetic studies show that AIF and endoG are involved in DNA fragmentation through the mitochondrial pathway because it is located within the mitochondria.^36^ ^37^

In the present study, it was found that low Bcl-2 expression increased 10.39 times the risk of PROM (OR = 10.39; 95% CI = 2.73–39.56; *p* = 0.001). A study by Harirah et al (2012)^14^ found that patients with spontaneous rupture of membrane demonstrate a higher expression of active caspase-3 and a lower expression of Bcl-2. In early pregnancy, apoptosis in the amniotic epithelium is independent of Bcl-2 activity, but at the end of pregnancy, it may have an important role in membrane weakness and rupture.^38^

A research by Fortunato et al (2001)^39^ on apoptosis and matrix metalloproteinase-2 (MMP-2) activation in PROM found that the increased expression and activity of MMP-2 is associated with PROM. A pro-apoptotic protein, such as p53, is produced in response to the fragmentation of DNA, binding to *MMP-2* gene promoters and leading to increased gene expression. This protein causes apoptosis by inducing the expression of the *BAX* pro-apoptosis gene and inhibiting *BCL2* anti-apoptosis gene expression. In PROM, pro-apoptotic genes expression (*BAX* and *p53*) are increased, whereas the anti-apoptotic *BCL2* gene expression is decreased.^10^ These findings are similar with our study, which suggests that Bcl-2 is an anti-apoptosis protein that regulates the apoptotic process in the fetal membrane, and its decrease is a risk factor for PROM.

The Bcl-2 family is a key regulator of apoptosis and is an essential component of the intrinsic pathway, regulating the permeability of the outer mitochondrial membrane and the release of pro-apoptotic factors, such as cytochrome C. In addition, members of the Bcl-2 family also connect both to the extrinsic and to the intrinsic pathways.^20^ The intrinsic pathway is centered in the mitochondria, with the Bcl-2 family as its main regulator. Its main action takes place in the outer membrane of the mitochondria, in which the apoptogenic factors are stored. Upon release, these apoptogenic proteins will activate caspase, as the executor of apoptosis.^20^ ^40^ ^41^

## Conclusion

Our findings suggest that apoptosis was involved in the mechanism of PROM. In patients at term pregnancy, caspase-dependent and -independent pathways of apoptosis were involved, evidenced by the high caspase-3 and AIF expressions, as well as by the low Bcl-2 expression. From the present study, we can conclude that a high expression of caspase-3 and AIF, and a decreased expression of Bcl-2, were risk factors for term PROM.
